# Relation of testosterone level and other factors with bone mineral density in male kidney transplant recipients: a cross-sectional study

**DOI:** 10.1186/s12882-023-03318-8

**Published:** 2023-09-14

**Authors:** Yasmine Salah Naga, Ola Atef Sharaki, Eman Zaki Azzam, Eman Mohamed Mostafa Farag, Montasser Mohamed Hussein Zeid

**Affiliations:** 1 Nephrology Unit, Internal Medicine Department, Faculty of Medicine, Alexandria University, Alexandria, Egypt; 2 Clinical and Chemical Pathology Department, Faculty Of Medicine, Alexandria University, Alexandria, Egypt; 3 Endocrinology Unit, Internal Medicine Department, Faculty of Medicine, Alexandria University, Alexandria, Egypt; 4 Internal Medicine and Nephrology Specialist, Alexandria University, Alexandria, Egypt

**Keywords:** Bone mineral density, Testosterone, Kidney transplantation

## Abstract

**Background:**

Although testosterone has a pivotal role in bone health, its correlation with bone mineral density (BMD) is understudied in kidney transplant recipients who are at high risk of osteoporosis. This study aimed to elucidate if there is any correlation between serum free testosterone and BMD in this population.

**Patients and methods:**

Sixty male kidney transplant recipients were enrolled in this cross-sectional study, and they were subjected to history taking, clinical examination, and laboratory investigations (including total and free testosterone). BMD was assessed in three regions (forearm, hip, and lumbar spine) using DEXA scan.

**Results:**

The mean age of the included patients was 45.55 ± 13.58 years. Serum total and free testosterone had mean values of 5.17 ± 1.4 ng/ml and 95.46 ± 28.24 pg/ml, respectively, with all levels within the normal range. DEXA scan detected osteoporosis and osteopenia in 9 (15%) and 30 (50%) patients in the lumbar region, 3 (5%) and 36 (60%) in the hip region, as well as 21 (35%) and 33 (55%) in the forearm region, respectively. BMD of the lumbar region had a significant positive correlation with free testosterone, phosphorus, and eGFR, while it had a significant negative correlation with platelets and patient age. BMD of the hip region was positively correlated with serum phosphorus, parathyroid hormone, and duration since the transplant, whereas it was negatively correlated with platelets and total testosterone level. BMD of the forearm had a significant positive correlation with eGFR, whereas it had a significant negative correlation with age and duration since transplantation. In addition, forearm BMD was significantly lower in patients with a radiocephalic AVF.

**Conclusion:**

Even within the normal range, free testosterone has a significant positive correlation with lumbar spine BMD with no significant association with the forearm or hip BMD.

## Introduction

Currently, kidney transplantation is the best management option for patients diagnosed with end-stage kidney disease (ESKD), as it is associated with a better quality of life and more prolonged survival compared to other management options [1]. However, bone affection is frequently encountered in kidney transplant recipients [2]. Bone mass is markedly reduced within the first year after transplantation [3, 4], which can lead to increased fracture risk in this population [5].

Multiple factors mediate the pathogenesis of that problem, including corticosteroid administration, calcineurin inhibitor intake, hypovitaminosis D, and hyperparathyroidism. The latter two could persist for a long period, even with a functioning renal graft [6]. These factors increase the fracture risk about three times in kidney transplant patients compared to patients receiving hemodialysis [7]. Not only do fractures increase morbidity after kidney transplantation, but they also increase health care costs and the risk of mortality [8–10]. Therefore, identifying the risk factors for that problem and its early management would enable physicians to improve the outcome of kidney transplant patients [11, 12].

Testosterone is the main sex steroid hormone in the male gender, and it is essential for the development of both primary and secondary sex characters [13]. Additionally, its role in maintaining bone mass and the prevention of osteoporosis has been established [14, 15]. Its beneficial action on human bones is mediated through stimulating both osteoblasts and chondrocytes via increasing the expression of different growth factors [16, 17] and inhibiting osteoclasts via the suppression of interleukin-6 [17, 18]. Male transplant patients are at increased risk of testosterone deficiency secondary to many factors including the immunosuppressive medications used and the effect of potential renal impairement on the hypothalamopitutary gonadal axis [19, 20].

Based on our intensive literature research, data connecting testosterone changes after kidney transplantation to bone mass changes are lacking. That provided a solid justification for us to carry out the current study, which examined the relationship between serum testosterone and bone mineral density (BMD) in male kidney transplant recipient more than one year following kidney transplantation.

## Patients and methods

This cross-sectional study was performed in the kidney transplantation units of both Alexandria Main University Hospital and Al Mouassah University Hospital after obtaining approval from the ethics committee of Alexandria University (Ethics Committee approval number 0201361). We conducted the study over a two-year period, from August 2020 to August 2022.

Our primary objective was to elucidate the relationship between serum free testosterone level and BMD using DEXA scan in male kidney transplant recipients, while the secondary objective was to study the correlation between BMD and other studied parameters.

Initially, we estimated the required sample size via the PASS software program, which highlighted the need for 60 participants to be included in order to detect the assumed positive correlation between serum free testosterone and BMD (with a 95% confidence interval and 2% precision using the interclass correlation test).

Our inclusion criteria were men aged between 18 and 60 years, having a kidney transplant at least 12 months prior to inclusion in the study to abolish the rapid bone loss in the first year of transplantation mentioned in previous studies [4, 21–23], and having an estimated glomerular filtration rate (eGFR) ≥ 60 ml/min/1.73m^2^ to minimize the sequelae of chronic kidney disease relate mineral bone disease (CKD-MBD). Additionally, we excluded patients who had a history of high dose glucocorticoid administration before the transplant procedure (defined as prednisolone more than 10 mg/day or equivalent dose for more than 3 months), who had rheumatologic disease requiring long term steroid administration or who were diagnosed with malignancy.

All patients signed an informed consent before participating in this trial. After that, they were subjected to detailed history taking focusing on the duration since kidney transplantation, the etiology of end-stage kidney disease, the presence of a radiocephalic arteriovenous fistula (AVF), other systemic medical comorbidities, and the commenced immunosuppressive therapy after transplantation.

Laboratory investigations included complete blood count (CBC), serum creatinine, corrected calcium, and phosphorus. Serum 25 hydroxyvitamin D, parathyroid hormone (PTH) and total testosterone level were estimated via electrochemiluminescence immunoassay (ECLIA). Serum free testosterone (pg/ml) was calculated [24].

BMD was assessed in all patients via dual-energy x-ray absorptiometry (DEXA) scan using GE Lunar dual-energy X-ray absorptiometry systems. BMD was measured in three regions; the hip, the lumbar spine, and the forearm. The measured T-scores were classified as normal (> -1 SD), osteopenia (-1 to -2.5 SD), or osteoporosis (< -2.5 SD) [25]. These values were recorded and correlated with the demographic and laboratory parameters.

The collected data were analyzed using the SPSS software version 27 for Windows® (IBM SPSS Inc, Chicago, IL, USA). Categorical data were expressed as numbers (with percentages). Quantitative data were presented as mean ± standard deviation (SD) if normally distributed and median and interquartile range (IQR) if non-normally distributed. The correlation between numerical parameters was evaluated using the Spearman correlation. Additionally, the Mann-Whitney test was applied to compare two groups of nonparametric data. Any p-value less than 0.05 on statistical analysis was considered significant.

## Results

### Demographic and clinical data

The mean age of the included patients was 45.55 years (± 13.58). The duration since transplantation had a median of 4 (1–8) years. Hypertension was the most common cause of ESKD before transplantation in 42 patients (70%), followed by diabetes mellitus in 12 patients (20%). Patients did not report a history of any pathological fractures or any alcohol use. They had been on hemodialysis for 19 ± 8.98 months prior to receiving live donor kidney transplantation.

Regarding the immunosuppressive medications, all patients were on oral prednisolone 5 mg/day, 42 patients (70%) were receiving mycophenolic acid, whereas tacrolimus was prescribed for 36 (60%) of them. Other medications included cyclosporin (24, 40%), mycophenolate mofetil (12, 20%), and everolimus (6, 10%). All included patients were not receiving oral calcium, vitamin D or bisphosphonates. Proton pump inhibitors (PPIs), specifically pantoprazole was used by only 3 patients (5% of our cohort). Radiocephalic AVF was present in 36 (60%) of the included participants (Table 1).

**Table 1:** Demographic and basic clinical and laboratory data in the study cases

	Study cases = 60
Age (Years)	45.55 ± 13.58
Duration since transplantation (Years)	4 (1–8)
Original kidney disease:	
Hypertension	42 (70%)
Diabetic kidney disease	12 (20%)
Unknown etiology	9 (15%)
Chronic glomerulonephritis	6 (10%)
Dialysis vintage before transplant (months)	19 ± 8.98
Medications	
Low dose corticosteroids	60 (100%)
Mycophenolic acid	42 (70%)
Tacrolimus	36 (60%)
Cyclosporin	24 (40%)
Mycophenolate mofetil	12 (20%)
Everolimus	6 (10%)
Radiocephalic AVF	36 (60%)
Laboratory data	
Hemoglobin (g/dl)	13.87 ± 0.73
WBCs X 10^9^ /L	7.46 ± 1.98
Platelets X 10^9^ /L	229 (162–553)
eGFR (ml/min/1.73m^2^)	85.55 ± 14.73
Creatinine (mg/dl)	1.15 ± 0.14
Corrected calcium (mg/dl)	9.15 ± 0.55
Phosphorous (mg/dl)	3.31 ± 0.63
Total testosterone (ng/ml)	5.17 ± 1.4
Free testosterone (pg/ml)	95.46 ± 28.24
Vitamin D (ng/ml)	16.44 (6.75–29.93)
PTH (pg/ml)	75.4 (32.10- 329.7)
Bone mineral density in the different regions:	
Bone mineral density (BMD) of lumbar region	-1.35 (-3.1 : 0.6)
Bone mineral density (BMD) of hip region	-1.20 (-2.7 : 0.7)
Bone mineral density (BMD) of forearm	-2.25 (-5.1 : 2.8)
Overall incidence of osteoporosis and osteopenia	
Osteoporosis at any site	27 (45%)
Osteopenia only at any site	27 (45%)
No osteoporosis/osteopenia at any site	6 (10%)

Categorical data presented as numbers (percentage). Quantitative data presented as mean ± SD if normally distributed and median (IQR) if non-normally distributed. BMD is expressed in mean (range)

### Laboratory data

The measured laboratory values of the study participants are shown in Table (1). All our patients had total and free testerone levels within the normal range (5.17 ± 1.4 ng/ml) and (95.46 ± 28.24 pg/ml), respectively.

### Bone mineral density

BMD of the lumbar region had a mean of -1.35 and ranged between − 3.1 and 0.6, whereas the mineral density of the hip region had a mean of -1.20 and ranged between − 2.7 and 0.7. In addition, the same parameter had a mean of -2.25 and ranged between − 5.1 and 2.8 in the forearm region. (Table 1).

**Fig. 1:**
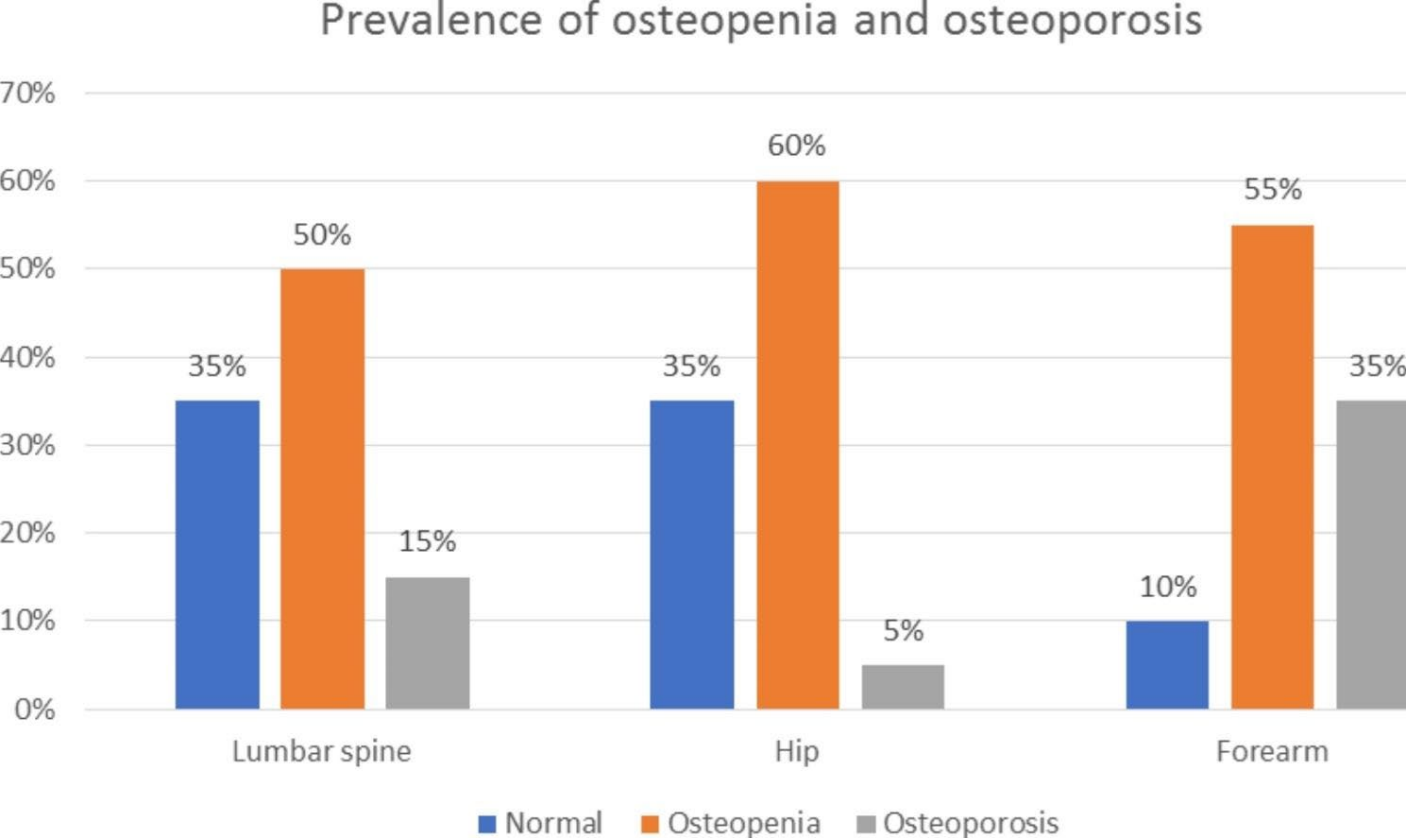
Prevalence of osteopenia and osteoporosis in the study population

Lumbar spine osteopenia and osteoporosis were detected in 30 patients (50%) and 9 patients (15%), respectively. In addition, the same two pathologies were encountered in 36 (60%) and 3 (5%) of the hips of the included patients, respectively, whereas in the forearm region, they were diagnosed in 33 (55%) and 21 (35%) patients, respectively (Fig. 1). Only two patients (3.33%) had combined osteoporosis affecting the forearm and lumbar spine. Overall, 27 (45%) patients had osteoporosis at any site, 27 (45%) had osteopenia alone at any site and only 6 (10%) patients did not have osteoporosis or osteopenia in all examined regions (Table 1).

BMD of the lumbar region had a significant positive correlation with free testosterone, phosphorus, and eGFR, while it had a significant negative correlation with platelet count and patient age. BMD of the hip region was positively correlated with serum phosphorus, PTH, and post-transplant duration, whereas it was negatively correlated with platelet count and total testosterone level. Lastly, BMD of the forearm had a significant positive correlation with total leukocytic count and eGFR, whereas it had a significant negative correlation with hemoglobin, age, and duration since transplantation (Table 2).

**Table 2:** Correlation between BMD of different bone regions with other clinical and laboratory data

		Bone mineral density (BMD) of the Lumbar region	Bone mineral density (BMD) of the hip region	Bone mineral density (BMD) Forearm
**Hemoglobin (g/dl)**	r_s_	-0.071	-0.066	**-0.289**
	p	0.588	0.618	0.025*
**WBCs (X 10** ^**9**^ **/L)**	r_s_	-0.020	0.026	**0.499**
	p	0.877	0.846	< 0.001*
**Platelets (X 10** ^**9**^ **/L)**	r_s_	**-0.477**	**-0.447**	0.167
	p	< 0.001*	< 0.001*	0.202
**Creatinine (mg/dl)**	r_s_	0.105	0.209	0.048
	p	0.424	0.108	0.717
**Corrected calcium (mg/dl)**	r_s_	-0.042	-0.213	0.081
	p	0.748	0.102	0.538
**Phosphorus (mg/dl)**	r_s_	**0.283**	**0.267**	-0.209
	p	0.028*	0.039*	0.109
**Testosterone total (ng/ml)**	r_s_	0.194	**-0.276**	-0.046
	p	0.138	0.033*	0.728
**Free testosterone (pg/ml)**	r_s_	**0.468**	-0.046	-0.044
	p	< 0.001*	0.728	0.741
**Vitamin D (ng/ml)**	r_s_	-0.044	-0.249	0.071
	p	0.736	0.055	0.587
**PTH (pg/ml)**	r_s_	0.160	**0.336**	-0.191
	p	0.223	0.009*	0.144
**Age (Years)**	r_s_	**-0.475**	-0.211	**-0.477**
	p	< 0.001*	0.105	< 0.001*
**Duration (Years)**	r_s_	-0.063	**0.271**	**-0.525**
	p	0.630	0.036*	< 0.001*
**eGFR (ml/min/1.73m** ^**2**^ **)**	r_s_	**0.263**	0.107	**0.415**
	p	0.042*	0.414	0.001*

*: Statistically significant (p < 0.05)

As shown in Table (3), patients with radiocephalic AVF expressed significantly lower BMD in the forearm (-2.55 vs. -1.4 in patients without radiocephalic AVF, p < 0.001). The presence of a radiocephalic AVF did not have a significant impact on lumbar or hip BMD.

**Table 3:** Comparison of BMD of different regions according to Radiocephalic AVF

	No Radiocephalic AVF (n = 24)	Radiocephalic AVF (n = 36)	Test of Significance
Bone mineral density (BMD) lumbar region	− 1.35 (− 2.90: -0.20)	− 1.35 (− 3.10: 0.60)	Z= -0.136 P = 0.892
Bone mineral density (BMD) hip	− 0.9 (-2.7: 0.4)	− 1.30 (− 2.30: 0.40)	Z= -0.544 P = 0.586
Bone mineral density (BMD) forearm	− 1.40 (− 2.10: 2.80)	-2.55 (-5.10 :-1.30)	Z= -5.987 P < 0.001*

Z: Mann-Whitney U-test

*: Statistically significant (p < 0.05)

## Discussion

Osteoporosis is a multi-factorial disease that may significantly impact patient health after transplantation. Although the impact of testosterone has been previously examined in transplant candidates before transplantation [26], its effect in kidney transplant recipients has not been studied. That is why we conducted the current study to elucidate the relationship between serum free testosterone and BMD after kidney transplantation.

Osteoporosis was present in 21 (35%) of the included forearms, 9 (15%) of the lumbar spine, and 3 (5%) of the included hips. Only two patients had combined forearm and lumbar spine osteoporosis (3.33%). Overall, only 6 (10%) of patients did not have osteoporosis or osteopenia in any site. The difference in the distribution of osteoporosis in the patients after kidney transplantation could be due to the difference in weight-bearing in the examined regions. Also, local factors like the presence of a radiocephalic fistula could play a role in the pathogenesis of that problem due to blood flow alterations [27]. This prevalence of bone disease lies within the reported rates in the literature, which reported a 50% incidence of osteopenia and a 15–56% incidence of osteoporosis during the follow-up of kidney transplant recipients [28].

In our study, we noted a significant positive correlation between free testosterone and lumbar BMD (r_s_ = 0.468 – p < 0.001) although all patients were within the normal range, while total testosterone did not have any significant correlation with the bone density of that region (p = 0.138). The beneficial impact of that hormone on bone is mediated through androgen receptors affecting the lineage of both osteoblasts and osteoclasts [29]. Similar to our results, Jørgensen and his associates reported a significant positive correlation between lumbar spine BMD and the bioavailable testosterone (β = 5.02, *P* = 0.002) in males prior to kidney transplant [26].

On the other hand, there was a negative correlation between total testosterone and hip BMD and no significant correlation between hip bone density and either free or total testosterone. Another study also reported no significant correlation between total or free testosterone and hip as well as forearm Z score among male hemodialysis patients [30]. In contrast, in the previously mentioned study conducted by Jørgensen et al., bioavailable testosterone had a significant positive correlation with total hip BMD in pre-transplant candidates (r_s_ = 6.35, P = 0.001). Nonetheless, total testosterone level did not have a significant correlation with the same parameter [26]. The lack of clear association in the hip and forearm region may be due to a larger role of local factors such as weight-bearing or the presence of an AVF in these regions.

In the current study, a significant negative correlation was noted between age and lumbar bone density (r_s_ = -0.475 – p < 0.001) as well as forearm bone density (r_s_ = -0.477 – p < 0.001) despite exclusion of elderly patients. No significant correlation was detected between age and hip BMD. Similarly, in a previous study, the authors reported a significant inverse correlation between age and lumbar spine BMD (r_s_ = -1.28 – p < 0.001), while it had no significant association with hip density (p = 0.14) [26]. Aging is normally associated with decreased bone formation, which could be explained by the shift from osteoblastogenesis to adipogenesis in the bone marrow. The latter has a toxic effect on bone mineralization and matrix formation [31].

The duration elapsed since transplantation had a significant positive correlation with hip BMD (r_s_ = 0.271, p = 0.036). Contrarily, it had a significant negative correlation with forearm BMD (r_s_ = -0.525 – p < 0.001) and no significant correlation with lumbar bone density. So, there is a heterogenicity of results regarding this parameter according to the tested bony region. Multiple previous studies reported no significant relationship between the time elapsed since transplantation and osteoporosis [32–34]. Nonetheless, two other studies highlighted the significant association between prolonged post-transplant periods and osteoporosis development [35, 36], probably because duration since transplantation is an indirect marker of the cumulative steroid dose and also corresponds with older age. These contradicting results may be a reflection of the interplay of different factors including different initial age of patients, different steroid doses and possibly pulse steroid administration along the transplantation course, different follow up periods and potential improvement of BMD after resolution of secondary hyperparathyroidism and CKD-MBD. We excluded elderly patients who may develop a more rapid decline in their BMD after transplantation and the duration since transplantation in our cohort was relatively short (median = 4 years). Inclusion of older patients and longer follow up may yield different results.

Our findings showed a significant negative correlation between platelet count and both lumbar and hip bone densities (p < 0.001). However, we did not notice any significant correlation between the same parameter and forearm bone density. No previous study has established this relationship in post-kidney transplant patients. Although the mechanism is not clear, a relation between platelet count and osteoporosis has been observed in multiple studies. Kim et al. reported that high normal platelet count was significantly detected in middle and old-aged patients with osteopenia and osteoporosis [37]. Another Swedish cohort by Kristjansdottir et al. [38] reported that high platelet count is associated with a decrease in bone mineral density. Akbal et al. also concluded that platelet functions have a strong relation to the bone mineralization, as platelet distribution width (PDW) and mean platelet volume (MPV) had a significant negative correlation with the development of postmenopausal osteoporosis [39].

Moreover, we did not detect any significant correlation between serum creatinine and BMD of either forearm, hip, or lumbar spine regions (p > 0.05), as also observed by Jørgensen et al. [26]. However, a study reported an inverse relationship between serum creatinine and BMD in post-transplant patients with poor graft function [34]. In contrast, our study showed a significant positive correlation between eGFR and both lumbar and forearm BMDs in agreement with Falkiewicz and his colleagues, who noted that patients with higher eGFR had higher BMD [40]. This is even though we only included patients with eGFR ≥ 60 ml/min/1.73m^2^, which highlights the value of eGFR over creatinine alone.

Regarding markers of CKD-MBD, we did not detect any significant correlation between corrected serum calcium and any of the measured BMDs (hip, lumbar spine, or forearm) in agreement with Mirfakhraee and his colleagues [30], which could be due to the fact that most of our participants had normal corrected serum calcium. However, there was a significant positive correlation between serum phosphorus and lumbar and hip BMDs in contrast to another study in kidney transplant recipients, which noted no significant difference in serum phosphorus levels among normal, osteopenic, and osteoporotic groups [32].

Vitamin D deficiency (serum level < 20 ng/ml) was present in 80% of the included participants, in accordance with a previous report where hypovitaminosis D was present in about 85% of adult kidney transplant recipients [41]. Still, we did not detect any significant correlation between vitamin D levels and the measured BMDs in either forearm, hip, or lumbar regions. A study in transplant recipients [35] and another in hemodialysis patients [42] also observed no significant association of vitamin D with bone density of any tested regions. On the contrary, other previous studies reported that vitamin D deficiency was associated with secondary hyperparathyroidism and decreased BMD in CKD and HD patients [43, 44]. As vitamin D is crucial for calcium absorption and bone mineralization, it should affect BMD [45], but impaired mineralization may occur at lower levels than those found in our cohort (16.49 ± 6.47 ng/ml).

Serum PTH had no significant correlation with both forearm and lumbar region BMDs. However, we detected a significant positive correlation between serum PTH and hip BMD. The association between PTH and BMD is heterogenous among studies, as some studies reveal a negative correlation between the two [26] [42, 46, 47], while others failed to show any correlation [48]. This heterogenicity may be attributed to different prevalence and different levels of persistent hyperparathyroidism after kidney transplant.

Interestingly, there was a significant decline in the BMD of the forearm in patients with a radiocephalic AVF (-2.55 vs. -1.4 in patients without AVF, p < 0.001). The presence of a radiocephalic AVF did not have a significant impact on the hip or lumbar bone densities. In line with our findings, Walder et al. reported that the measured T-score was significantly decreased in the radius of patients with previous AVF [49]. Multiple theories have been proposed to explain the decreased BMD in patients with AVF, including changes in blood flow, increased sympathetic tone, and the effect of AVF pulse pressure on bone turnover. These factors could induce bone loss in the forearm region in a similar way to the effect of abdominal aortic aneurysms on the related vertebrae [50–52]. Additionally, immobilization and underuse of the AVF-containing limb could play a role [53, 54].

Although our study is one of the first studies to assess the effect of testosterone on bone health in male kidney transplant recipients, it still has some limitations. It included a relatively small sample of patients. Other factors which may impact BMD such as level of activity and weight-bearing, PPI use, magnesium levels, pre-transplant markers of CKD-MBD and family history of osteoprosis were not assessed. In addition, the lack of patients with low testosterone in our cohort may have attenuated the results. In addition, the lack of heterogeneity in the sample due to the choice of only adult males prevents generalization of the conclusions. Therefore, the factors contributing to osteoporsis following kidney transplantation need further exploration in studies, including larger and more heterogeneous samples.

## Conclusion

Free testosterone had a significant positive correlation with lumbar spine BMD with no significant association with the forearm or hip BMD. The presence of radiocephalic AVF is significantly associated with a decrease in forearm BMD. Osteopenia and osteoporosis are not uncommon and are often overlooked in males post-transplantation, therefore, screening in high risk patients is advised.

## Data Availability

All data generated during this study are included in this published article and all datasets used and/or analysed are available from the corresponding author on reasonable request.

## List of Abbreviations

AVF: Arteriovenous fistula
BMD: Bone mineral density
CBC: Complete blood count
CKD: Chronic kidney disease
DEXA: Dual-energy X-ray absorptiometry
ECLIA: Electrochemiluminescence immunoassay
eGFR: Estimated glomerular filtration rate
ESKD: End stage kidney disease
HD: Hemodialysis
IQR: Interquartile range
MBD: Mineral and bone disorder
PDW: Platelet distribution width
PPI: Proton pump inhibitor
PTH: Parathyroid hormone
SD: Standard deviation
SPSS: Statistical package of social sciences
